# Fulguration of Benign Papilloma of Bladder

**Published:** 1915-10

**Authors:** 


**Fulguration of Benign Papilloma of Bladder.** Herman L. Kretschmer of Chicage, Interstate Med. Jour., Sept., reviews 49 articles in the literature. The immediate results are successful and few complications have been reported. Apparent cures have even been reported for carcinoma.

